# Optimizing the Teachable Moment for Health Promotion for Cancer Survivors and Their Families

**Published:** 2016-05-01

**Authors:** Melissa L. Frazelle, Patricia J. Friend

**Affiliations:** Loyola University Chicago, Marcella Niehoff School of Nursing, Chicago, Illinois

Due to significant advances in screening, detection, and treatment, 70% of patients with cancer are living 5 years or longer, comprising 4% of the US population (Demark-Wahnefried et al., 2015). As of January 2014, the American Cancer Society (ACS) had estimated that there were approximately 14.5 million cancer survivors in the United States, and that this number is expected to increase to approximately 19 million by 2024 (ACS, 2014; see Figure 1).

**Figure 1:**
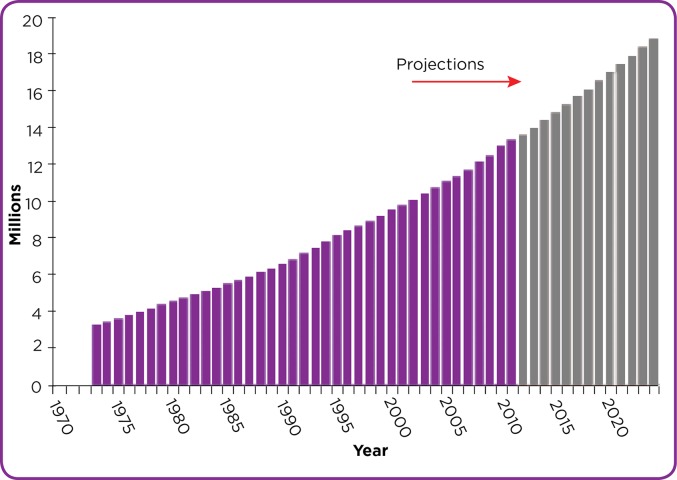
Estimated number of cancer survivors in the United States. Information from the National Cancer Institute. Adapted from www.cancer.gov/ (2016).

The growing cancer survivorship population is also impacted by the increase in the older adult population aged 65 years and older; this population, which is the most susceptible to cancer, is expected to double by the year 2030 (Institute of Medicine [IOM], 2013). Therefore, as the number of adult cancer survivors grows, long-term cancer survivorship care and follow-up are going to fall on advanced practitioners (APs) to bridge the gap between oncology and primary care physicians (Furlow, 2015).

The IOM report, "From Cancer Patient to Cancer Survivor: Lost in Transition," was one of the first documents to propose key recommendations to improve the quality of survivorship care. The IOM defines survivorship care as the period of care following completion of primary treatment (IOM, 2006). A cancer "survivor" is defined as an individual with cancer from the time of diagnosis through the balance of his or her life (IOM, 2006). Many guidelines refer to 5 years posttreatment as the official survivorship mark; however, for the purposes of this article, survivorship is considered the time after completion of active treatment.

Cancer survivors and their providers are concerned with cancer recurrence and secondary cancers due to survivors’ increased risk compared with the general population (Weaver, Jessup, & Mayer, 2013). Cancer survivors also have a higher risk of developing comorbid conditions, such as dyslipidemia, obesity, diabetes, premature menopause, decreased bone mass, hypertension, and hypothyroidism (Edgington & Morgan, 2011).

Despite similar lifestyle behaviors, research has found that individuals with a history of cancer have a higher prevalence of chronic conditions, even when compared with age- and sex-matched controls (Berry et al., 2014). In fact, the majority of cancer survivors have at least one comorbid condition, and many adult cancer survivors die of chronic disease, such as cardiovascular disease, stroke, or diabetes complications, rather than cancer itself (Ganz, 2011; Wolin, Dart, & Colditz, 2013). Therefore, survivors are not only trying to lead normal healthy lives free of recurrence, but also free of chronic diseases (IOM, 2013).

Recent Survivorship Guidelines from the National Comprehensive Cancer Network (NCCN, 2015b) focus on late and long-term effects, as well as preventive risk-reduction strategies via lifestyle changes. This approach represents an expanded focus from mainly disease surveillance to encompass active risk reduction via modifiable lifestyle changes (Beck & Mooney, 2015; Edgington & Morgan, 2011; Taylor & Monterosso, 2015).

## THE TEACHABLE MOMENT FOR PATIENTS AND FAMILY MEMBERS

The nature of the survivorship period presents a unique opportunity for patients with cancer and their families, because overcoming cancer induces a trauma-like experience and disrupts the lives of patients and their families (Bell, 2012). This period after active treatment is considered a "teachable moment," the time frame following a health event in which a patient is most conducive to lifestyle changes. Research suggests that cancer survivors are often highly susceptible to messages about the role of lifestyle during the survivorship period and tend to see this as a way of being in control over the possibility of recurrence (Bell, 2012). The literature also suggests that cancer survivorship can be considered a teachable moment for families because of the shared experience (Breitkopf et al., 2013). Not only are family members dealing with the stress of a loved one having cancer, many now have the burden of being a caregiver. In addition, many family members may have genetic or environmental risk factors they need to consider, further motivating them to lead healthier lives during this time (Beck & Mooney, 2015). Therefore, survivorship is a crucial time to transform the lifestyle behaviors of patients and families and maximize their chances of avoiding recurrence of cancer as well as developing primary cancers/secondary cancers and chronic diseases.

Multiple studies have found that patients and family members of cancer survivors express strong intentions to engage in health-promoting behaviors related to physical activity and nutrition during the posttreatment transition (Mazanec, Flocke, & Daly, 2015), with interest in participating in risk reduction and family-based health promotion programs (Breitkopf et al., 2013; Cooley et al., 2013). Heightened receptivity to make healthy behavior changes makes this an ideal time to introduce and deliver evidence-based interventions aimed at modifiable risk factors (Bell, 2012). "Teachable moments" do not automatically lead to behavior change; rather they need to be a deliberate and intentional part of the patient teaching and consultation. Advanced practitioners need to optimize this underused window of opportunity to employ family-based strategies to encourage health promotion interventions (Mazanec et al., 2015).

## HEALTHY SURVIVORSHIP

As the general population ages and the cancer survivor population increases, there is an urgent need for increased attention and greater coordination of health promotion and disease prevention in survivors (McCabe et al., 2013). The ACS identifies the top four modifiable risk factors for cancer prevention and recurrence as smoking, weight control, dietary choices, and levels of physical activity (Kushi et al., 2012). The American Institute for Cancer Research (2015) projects that one-third of the most common cancers in the United States are preventable through a healthy diet, physical activity, and weight management. Another third of cancers are attributable to exposure to tobacco products (Kushi et al., 2012). Thus, approximately two-thirds of US cancer cases are preventable each year, making lifestyle modifications imperative to cancer prevention and overall survival (Lanou & Svenson, 2011).

The most updated ACS Guidelines on Nutrition and Physical Activity for Cancer Survivors (2012) addresses these modifiable risk factors, noting the three main health promotion recommendations for cancer survivors as achieving and maintaining a healthy weight; engaging in regular physical activity; and having a dietary pattern that is high in vegetables, fruits, and whole grains (Rock et al., 2012). The ACS guidelines are congruent with healthy lifestyle recommendations for nonsurvivors and can be applied to family members to optimize health by decreasing their risk of chronic disease and primary cancers.

**Smoking Cessation**

The ACS Guide to Quitting Smoking supports that smoking cessation is the single most important step smokers can take to enhance the length and quality of their lives (ACS, 2016). Thus, smoking cessation should be a priority for all cancer survivors and family members, just like the general population (Ligibel, 2012; NCCN, 2015a).

Smoking is an established risk factor for lung, esophageal, and head and neck cancers (Cooley et al., 2013; Ligibel, 2012). A recent meta-analysis demonstrated that cancer survivors who continue to smoke have an increased risk of recurrence and all-cause mortality (Ligibel, 2012). Smoking has also been found to reduce quality of life and increase treatment side effects, as well as increase survivors’ risk of chronic diseases such as cardiovascular disease, stroke, and osteoporosis (Wolin et al., 2013). Moreover, smoking cessation has been shown to lower the risk of new primary tumors, metastases, and recurrence, and it can also improve overall treatment response and decrease treatment toxicities (Wolin et al., 2013). Nonsmoking adults whose lifestyles were most consistent with the ACS Guidelines on Nutrition and Physical Activity for Cancer Prevention had a significantly lower risk of dying from cancer, cardiovascular disease, and all causes combined (Kushi et al., 2012). Therefore, it is imperative that survivors and their family members understand the harms of smoking and be encouraged to quit (Karam-Hage, Cinciripini, & Gritz, 2014).

**Diet and Nutrition**

In the United States, more than one-third of cancer deaths each year are linked to poor diet and sedentary lifestyle (Kushi et al., 2012). Dietary choices are essential for healthy survivorship. The ACS recommends that survivors follow the ACS Guidelines on Nutrition and Physical Activity for Cancer Prevention. It involves limiting the consumption of processed and red meat (and choosing lean meat and/or fish high in omega-3 instead), eating at least 2.5 cups of vegetables and fruits every day, choosing whole grains that are high in fiber instead of refined products, avoiding processed foods, choosing unsaturated fats over saturated and trans-fats, and limiting consumption of alcohol to 1 drink daily for women and 2 drinks daily for men (Demark-Wahnefried et al., 2015).

These recommendations are based on substantial research demonstrating that a plant-based diet high in fruits, vegetables, whole grains, and some poultry and fish is associated with reduced cancer survivor mortality compared with the "Western" diet, which is high in refined grains, processed and red meats, sugars, and high-fat dairy products (Demark-Wahnefried et al., 2015; Rock et al., 2012). Moreover, substantial evidence suggests that the adoption of a vegan or vegetarian diet can be a useful strategy for cancer prevention, recurrence, and chronic disease (Lanou & Svenson, 2011; McBride, 2015).

A lower intake of saturated and trans-fat in the postdiagnosis diet has been associated with improved overall survival for patients with breast cancer (Beasley et al., 2010). High-fat dairy consumption has been associated with an increased risk of all-cause mortality after breast cancer diagnosis (Kroenke, Kwan, Sweeney, Castillo, & Caan, 2013; Wolin & Colditz, 2013).

High sugar intake has not been proven to directly increase the risk for progression of cancer. However, high sugar intake favors weight gain and diabetes, which is associated with an increased risk of cancers (Rock et al., 2012).

**Physical Activity**

The ACS guidelines recommend avoiding inactivity and returning to normal daily activity as soon as possible after a cancer diagnosis. They also suggest survivors follow recommendations for the general population per the US Department of Health and Human Services, including doing aerobic exercise of moderate intensity for at least 150 min/week in divided sessions or engaging in 75 minutes of vigorous exercise or an equivalent combination of both. Resistance strength-training exercise for at least 2 days/week is also recommended for bone health (Rock et al., 2012).

At least 20 observational research studies have shown that higher levels of physical activity can improve overall mortality, lower the risk of cancer recurrence, and improve survival among a range of survivor diagnoses including breast, colorectal, prostate, and ovarian cancers (Rock et al., 2012). The combination of aerobic exercise and resistance training is associated with a lower risk and better control of cardiovascular disease, osteoporosis, and diabetes in the general population, and this translates to the cancer survivorship population (Demark-Wahnefried et al., 2015). Even light-intensity physical activity, such as walking 2.5 miles/hour, has been found to have a favorable association with various biomarkers that are linked to cancer and other chronic diseases (such as hyperlipidemia, diabetes, heart disease, and obesity), including white blood cell counts (WBCs), neutrophils, and insulin resistance (Loprinzi & Lee, 2014).

Vigorous exercise is a newer recommendation from a recent Cochrane database systematic review of 56 trials (Lemanne, Cassileth, & Gubili, 2013). The review demonstrated that including high-intensity aerobic activity, such as jogging, running, and calisthenics, might result in more metabolic benefits than light-intensity aerobic activity, by improving adiposity, insulin resistance, and inflammation (Lemanne et al., 2013). This review also suggests that higher-intensity exercise has greater benefits than low-intensity workouts related to quality of life and treatment side effects. Therefore, if cancer survivors are not already active, they should engage in light-intensity physical activity; and if cancer survivors do not have any contraindications and are active, they should add some vigorous activity to their base of moderate-intensity activity.

**Weight Management**

Maintaining a healthy weight is relevant to survivors and family members, considering obesity is an epidemic in the United States and a well-established risk factor not only for cancer, but for chronic diseases (Rock et al., 2012). Being overweight and being obese are both acknowledged as increasing the risk of breast, colon, endometrial, gastric, kidney, and pancreatic cancers. Simply being overweight increases survivors’ risk of recurrence and reduces the likelihood of overall survival (Rock et al., 2015).

According to caloric restriction research, reduction of caloric intake and energy restriction reduces the risk of cancer in overweight and obese patients (Albini, Tosetti, Li, Noonan, & Li, 2012). Like physical activity, weight-loss interventions also demonstrate improvements in biomarkers linked to cancer risk and outcomes (Demark-Wahnefried et al., 2015). Therefore, weight reduction is recommended for overweight/obese cancer survivors and their family members to decrease recurrence and the risk of cancer.

The ACS weight management guidelines recommend maintaining a healthy weight and a body mass index (BMI) between 18.5 and 24.9 kg/m₂ (Edgington & Morgan, 2011). If a person is overweight or obese, the ACS recommends limiting consumption of energy-dense foods and beverages and increasing physical activity to promote weight loss (Demark-Wahnefried et al., 2015). Research suggests that even if an ideal weight is not achieved, it is likely that any weight loss through healthy eating and exercise is beneficial, with weight losses of 5% to 10% likely having significant health benefits for cancer survivors, just as for the noncancer population (Rock et al., 2012).

## CURRENT HEALTH BEHAVIORS IN SURVIVORS

Despite evidence demonstrating the benefits of positive lifestyle practices, a significant portion of cancer survivors do not meet ACS lifestyle guidelines and are inactive, have a poor diet, and/or are obese. Research suggests that many of these negative health behaviors are present at the time of diagnosis and may become even more evident after treatment (Demark-Wahnefried et al., 2015).

The prevalence of smoking among cancer survivors is similar to that of the US general population, with smoking being the highest among young adult survivors and twice as high among lung cancer survivors (Cooley et al., 2013; Wolin et al., 2013). Risk factors independently associated with persistent smoking in survivors are being female, low income, high-risk alcohol use, high BMI, presence of household members who smoke, and longer duration of smoking (Kim, Kim, Park, Shin, & Song, 2015). Cancer survivors without health insurance also have substantially greater smoking rates than those with health insurance (Burcu, Steinberger, & Sorkin, 2015).

Approximately 70% to 80% of cancer survivors are relatively inactive and do not engage in sufficient exercise (Loprinzi & Lee, 2014). Survey data noted that cancer survivors aged 20 to 64 years old have a higher prevalence for overweight/obese status, not meeting physical activity guidelines, and current smoking status than controls (Nayak, Paxton, Holmes, Nguyen, & Elting, 2015). One study found that African American and Hispanic survivors were more likely to have a higher BMI than white survivors; African American survivors were less likely to meet physical activity recommendations; and Native Americans and multiracial survivors were more likely to be current smokers than non-Hispanic white survivors (Nayak et al., 2015). African Americans, women with less education, and older women were more likely to be obese and have more comorbidities (Highland, Hurtado-de-Mendoza, Stanton, Dash, & Sheppard, 2015). Thus, there are researched disparities in adoption of healthy lifestyle behaviors (O’Neill et al., 2013).

On the other hand, there has been some evidence that cancer survivors are making lifestyle changes. In the 2010 LIVESTRONG survey, the majority of survivors reported they made healthy dietary changes and participated in regular physical activity (Low et al., 2014). However, the survey was voluntary and relied on self-reporting, so it might not represent the experiences of all survivors. The LIVESTRONG survey did note important factors associated with greater likelihood of engaging in positive lifestyle behaviors, including higher education, greater knowledge about how to reduce cancer risk, and reporting of more positive emotional benefits in the cancer experience (Low et al., 2014).

## CANCER SURVIVOR AND FAMILY NEEDS

A cross-sectional study of 255 survivors revealed that approximately 63% of cancer survivors reported one or more unmet needs (Willems et al., 2015). The ACS’s Survey of Cancer Survivors II looked at cancer survivors’ unmet needs and found that all survivors were concerned about recurrence and cited a need for more cancer-related information and education (Kaplan, 2015). Another study also found that survivors want more information regarding health-promotion techniques involving diet, exercise, and weight management (James-Martin, Koczwara, Smith, & Miller, 2014).

Survivors and their family members indicate strong intentions to engage in lifestyle changes related to physical activity and nutrition during the posttreatment transition (Mazanec et al., 2015). In the pilot study of lung cancer survivors and their families, readiness to change behaviors within the next 6 months ranged from 63% for physical activity, 73% for diet, and 88% for smoking cessation for survivors and 81% for physical activity, 58% for diet, and 91% for smoking cessation for family members (Cooley et al., 2013).

## INTERVENTIONS TO SUPPORT POSITIVE HEALTH BEHAVIOR

Evidence-based interventions to address patients’ needs as well as decrease recurrence and comorbidities are evolving considerably, as more studies demonstrate that positive lifestyle behaviors are associated with increased survival from cancer, other medical conditions, and improvement in overall quality of life (Beck & Mooney, 2015). Although some cancer survivors know that healthy lifestyle behaviors play a role in reducing recurrence and comorbidities, their awareness does not always translate into action (O’Neill et al., 2013). Therefore, practical health-promotion interventions need to be tailored to patient needs, circumstances, and goals. Evidence suggests that the largest benefits in changing patients’ activity behavior come from face-to-face sessions, supervised exercise sessions, clear and challenging exercise goals, behavior-modification techniques, intensive and ongoing contact, written materials to supplement advice, and individual tailoring of interventions.

A recent systematic review and meta-analysis of 14 randomized controlled trials found that highly structured exercise interventions tended to produce the largest behavior change overall, and the largest benefits resulted from interventions supported by phone counseling or e-mail (Bluethmann, Vernon, Gabriel, Murphy, & Bartholomew, 2015). Another systematic review also showed that diet, exercise, and smoking cessation were consistently changed with in-person interventions, including phone and mail contact (Green, Hayman, & Cooley, 2015).

**Figure 2:**
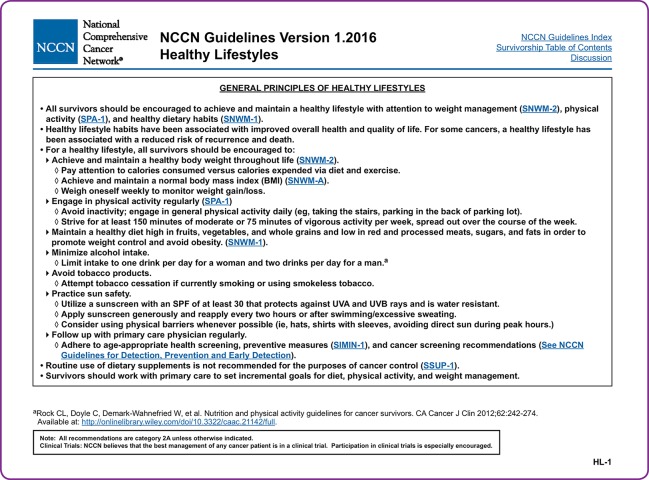
Reproduced with permission from the NCCN Clinical Practice Guidelines in Oncology (NCCN Guidelines®) for Healthy Lifestyles Version 1.2016. © 2016 National Comprehensive Cancer Network, Inc. All rights reserved. The NCCN Guidelines® and illustrations herein may not be reproduced in any form for any purpose without the express written permission of the NCCN. To view the most recent and complete version of the NCCN Guidelines, go online to NCCN.org. NATIONAL COMPREHENSIVE CANCER NETWORK®, NCCN®, NCCN GUIDELINES®, and all other NCCN Content are trademarks owned by the National Comprehensive Cancer Network, Inc.

Focusing on survivors’ specific health goals could be an effective strategy to help survivors achieve their personal goals, as well as accounting for age, gender, socioeconomic, racial and ethnic, and comorbidity type differences surrounding the adoption of recommended health behaviors (Highland et al., 2015; LeMasters, Madhavan, Sambamoorthi, & Kurian, 2014; Nayak et al., 2015; Palmer, Bartholomew, McCurdy, Basen-Engquist, & Naik, 2013). Thus, a single approach to implementing health behaviors does not work for every patient, and multiple factors need to be considered when creating practical interventions for patients and their families (Boyajian et al., 2014). See Table 1 for sample strategies to promote healthy lifestyles and Figure 2 for general principles of healthy lifestyles for cancer survivors that should be recommended.

**Table 1:**
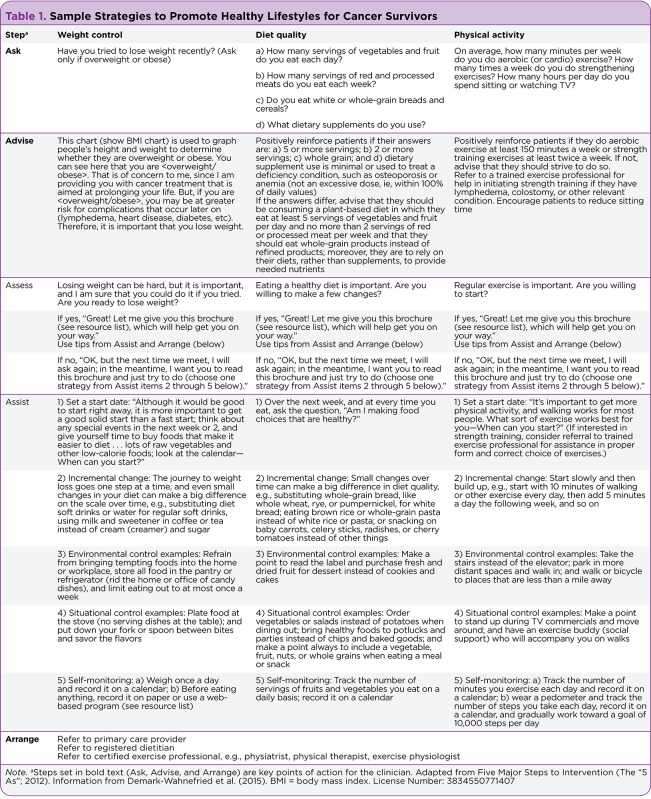
Sample Strategies to Promote Healthy Lifestyles for Cancer Survivors

**Health Promotion/Wellness Programs**

Health promotion/wellness programs specifically for cancer survivors are underway, yet research to identify the most effective means of promoting health-behavior change among cancer survivors is not established (Buchanan, Houston, & Richardson, 2015).

The FRESH START trial, a randomized controlled study of 543 patients with colon and breast cancers, established that durable lifestyle changes in diet and exercise are feasible, as adherence to behavior changes 1 year after the intervention was noted (Demark-Wahnefried et al., 2015). Results of the Exercise and Nutrition to Enhance Recovery and Good Health for You (ENERGY) Trial found that behavioral weight-loss interventions can lead to clinically meaningful weight loss in overweight/obese survivors and can have favorable effects on physical activity and blood pressure (Rock et al., 2015). Two large randomized trials, Women’s Intervention Nutrition Study (WINS) and the Women’s Health Eating and Living (WHEL) study, found that a low-fat diet reduced the risk of recurrence and mortality in women with breast cancer in the WINS study and resulted in weight loss in both trials (Ligibel, 2012).

A systematic review of 14 randomized controlled trials demonstrated evidence that exercise interventions for cancer survivors can improve aerobic exercise tolerance at 8 to 12 weeks and even after 6 months. The review suggested that exercise behavior programs should be tailored to the survivor’s capabilities, including frequency, duration and intensity of sets, repetitions, and intensity of resistance training, to be most effective (Bourke et al., 2014).

Evidence exists that smoking cessation programs addressing multiple health behaviors have been associated with successful behavioral change in survivors and family members (Cooley et al., 2013; see Table 2). A recent study found that survivors’ perceptions of the risks of smoking on their prognosis, knowledge of the severity of health problems from smoking, cessation barriers, and the benefits of quitting are appropriate targets for interventions for survivors continuing to smoke (Westmaas, Berg, Alcaraz, & Stein, 2015). Patient engagement is crucial for optimal results for quit rates (Burcu et al., 2015; See Table 2 for the "5 As" of Brief Intervention for Smoking Cessation).

**Table 2:**
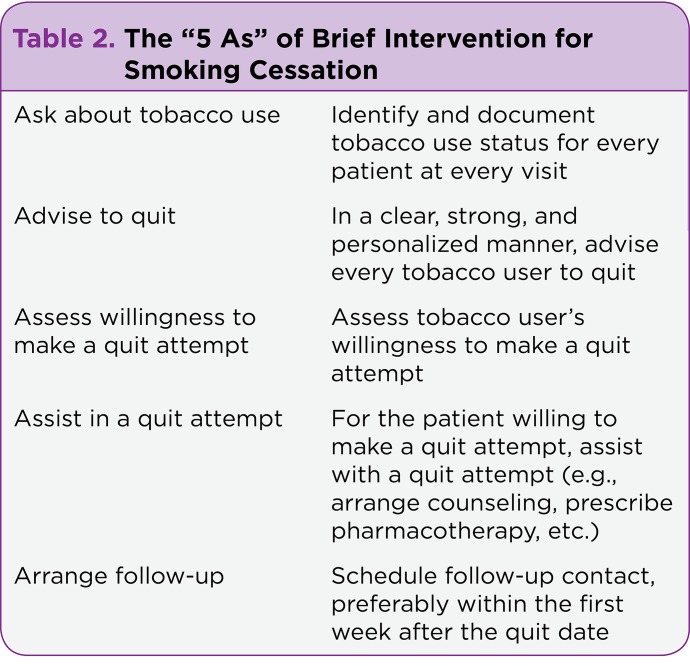
The "5 A’s" of Brief Intervention for Smoking Cessation

**Family Influence**

Targeting survivors’ family members may enhance adoption of healthy behaviors, because cancer is a shared experience and health behaviors tend to cluster within families (Cooley et al., 2013). Family members can have a positive influence on survivors’ health behaviors, including weight-loss behaviors, smoking cessation, and engagement in physical activity (Mazanec et al., 2015). In addition, positive behavior change in one spouse has been observed to create similar change in the other (Mazanec et al., 2015). Thus, survivorship is an opportunity to increase awareness, perceptions of vulnerability to cancer, and receptivity to cancer risk–reducing behaviors for cancer survivors and their families (Breitkopf et al., 2013).

**AP Involvement**

Promoting positive health behaviors, including lifestyle changes, can minimize recurrence and health risks associated with chronic disease (Low et al., 2014). The power of suggestion by health-care providers regarding lifestyle changes, including exercise and diet, has been underscored, and it has been documented that patients are much more likely to engage in healthy behaviors if they are recommended by a provider (Rock et al., 2012). One study found that a physician’s simple recommendation to exercise after cancer treatment resulted in a significant improvement in the number of hours the patient exercised per week (Demark-Wahnefried et al., 2015). Thus, involvement by APs is essential for promoting and overseeing healthy lifestyle adaptations.

Oncologists and APs know there is evidence linking lifestyle behaviors to cancer recurrence, yet one study found that cancer specialists do not always emphasize health-promotion behaviors, unless the evidence is substantial (Coa et al., 2015). On the other hand, primary care providers view health promotion as being important to all patients (Coa et al., 2015). Therefore, as evidence grows regarding favorable lifestyle changes and cancer recurrence/prognosis, it is important that this information is communicated to oncology specialists, primary care providers, and APs.

Lastly, from an economic perspective, motivating survivors and their family members to adhere to positive health behaviors is cost-free to endorse and monitor. Where value is lacking in intensive surveillance for recurrence, real value could be added to survivorship care by monitoring and reinforcing health behaviors for survivors and their families (Beck & Mooney, 2015).

## CARE COLLABORATION

Primary care providers and oncology specialists have been identified as providers of wellness care and specialized cancer care, respectively (Hebdon, Fahnestock, & McComb, 2015). However, collaboration by both is variable due to the fragmented nature of the health-care system and challenges with rolling out systems such as the survivorship care plan (SCP; Furlow, 2015; Ganz, 2011). A systematic review noted that care continuity is a common theme among survivors, and increased coordination and clarity of provider roles are needed to effectively monitor survivors’ health behaviors post treatment (Hebdon et al., 2015). The SCP can serve as a vehicle for survivors to focus on healthy lifestyles and coordinate communication between providers.

The American College of Surgeons, Commission on Cancer, developed a SCP standard (Standard 3.3) that requires accredited cancer centers "to develop and implement a process to disseminate a treatment summary and follow-up plan to patients who have completed cancer treatment" (American College of Surgeons, 2016). This standard was implemented in January 2015, and by the end of 2016, at least 25% of eligible patients who have completed treatment must be provided with a SCP by accredited centers. Specifically, survivors should work with primary care providers to set goals for diet, physical activity, and weight management (NCCN, 2015b).

## IMPLICATIONS FOR APS

Advanced practitioners can ease the transition for survivors and their family members from active treatment to follow-up care (Hebdon et al., 2015). There are promising data for APs managing care of survivors and their families. A study of 759 survivors of breast cancer found that survivorship visits with an oncology advanced practice nurse (APN) correlated with more discussions of lifestyle modifications when compared with visits by oncologists or primary care physicians, but they all had comparable screening rates (Kenison, Silverman, Sustin, & Thompson, 2014). Advanced practitioners should focus on survivors’ and family members’ goals for healthy lifestyle behaviors and consider differences in individual needs and identified disparities in health behaviors when creating health-promotion interventions. Structured evidence-based interventions that are tailored to survivors and their family should be offered with adequate follow-up to ensure adherence (see Tables 1 and 2, and Figure 2).

## CONCLUSION

More well-designed, large, randomized, and controlled intervention trials are needed to test the direct impact of physical activity, weight management, dietary interventions, and smoking cessation on the quantity and quality of life of cancer survivors. Nonetheless, now is the time to bring health promotion into cancer survivorship practice. The general consensus is that healthy survivorship has the greatest impact when multiple risk-reduction behaviors work in synergy. Thus, more research needs to be conducted to better understand successful multiple behavioral risk-reduction interventions that facilitate sustainable lifestyle changes in both survivors and family members over time. We have a poor understanding of how to effectively encourage survivors to meet exercise and dietary guidelines, so further research is needed to investigate diet and physical activity intervention details. Lastly, addressing the needs of family members of survivors and how family-based health-promotion strategies affect survivors’ health is understudied and an important area for further research as well. Survivors, their families, and the survivors’ APs will all benefit from more evidence-based research on how best to promote and maintain healthy lifestyles in cancer survivors.

The cancer trajectory is a life-changing event for patients and their families. The posttreatment transition offers an opportune time for survivors to seriously contemplate making lifestyle changes that have potential to decrease recurrence, primary/secondary cancers, and chronic disease. Provider recommendations and follow-up of health-promoting behaviors are key during this time to increase engagement and monitor adherence. Health promotion/wellness programs should be offered to patients, and evidence suggests that patient-family interventions may be even more successful. Survivors’ and families’ health-promotion goals should be assessed and recommendations individualized based on specific needs and health-promotion goals. Tailored interventions for survivors, based on age, gender, socioeconomic status, ethnic and racial differences, and comorbidity type, may be most effective when accounting for factors that impact health promotion engagement and adherence. Although such individualized plans would have an economic cost, the long-term savings of preventing cancer recurrence, other cancers, and comorbidities would be substantial.

In addition, highly structured evidence-based interventions with strong follow-up and patient-provider communication through in-person visits, phone calls, and e-mails should be implemented to enhance outcomes. Better collaboration between oncology and primary care providers is also necessary to improve the continuity of care and communication of survivor and family health-promotion goals. Advanced practitioners are well suited to provide survivorship care and bridge the gap between oncology and primary care providers. Lastly, evidence-based research regarding successful health-promotion interventions, including SCPs, and cancer recurrence needs to be dispersed among oncology, primary care physicians, and APs to increase health-promotion discussions with survivors. Treatment success is a short-term victory if patients die from recurrence, secondary malignancy, or chronic disease. Thus, optimizing the teachable moment for health promotion through successful interventions is a key to overall survival.
